# Supplementary material for: Investigations of Single-Subunit tRNA Methyltransferases from Yeast
Source: J Fungi (Basel). 2023 Oct 19;9(10):1030. doi: 10.3390/jof9101030 (PMC10608323; doi:10.3390/jof9101030)
Supplement: Supplementary file 1 [file jof-09-01030-s001.zip › jof-2609251-supplementary.pdf]

```
Trm1p     1  .......MEGFFRIP..LKRANLHGMLKAAIS...KIKANFTAYGAPRINIEDFNIVKEG
Trmt1p    1  MQGSSLWLSLTFRSARVLSRARFFEWQSPGLPNTAAMENGTGPYGEERPREVQETTVTEG

Trm1p    49  KAEILFPKKETVFYNPIQQFNRDLSVTCIKAWDNLYGEECGQKRNNKKSKKKRCAETNDD
Trmt1p   61  AAKIAFPSANEVFYNPVQEFNRDLTCAVITEFARIQLGAKGIQIKVPGEKDTQKVVVDLS

Trm1p   109  SSKRQ........KMGNGSPKEAVGNSNRNEPYINILEALSATGLRAIRYAHEIPHVREV
Trmt1p  121  EQEEEKVELKESENLASGDQPRTAAVGEICEEGLHVLEGLAASGLRSIRFALEVPGLRSV

Trm1p   161  IANDLLPEAVESIKRNVEYNSVENIVKPNLDDANVLMYRNKATNNKFHVIDLDPYGTVTP
Trmt1p  181  VANDASTRAVDLIRRNVQLNDVAHLVQPSQADARMLMYQHQRVSERFDVIDLDPYGSPAT

Trm1p   221  FVDAAIQSIEEGGLMLVTCTDLSVLAGNGYPEKCFALYGGANMVSHESTHESALRLVLNL
Trmt1p  241  FLDAAVQAVSEGGLLCVTCTDMAVLAGN.SGETCYSKYG.AMALKSRACHEMALRIVLHS

Trm1p   281  LKQTAAKYKKTVEPLLSLSIDFYVRVFVKVKTSPIEVKNVMSSTMTTYHCSRCGSYHNQP
Trmt1p  299  LDLRANCYQRFVVPLLSISADFYVRVFVRVFTGQAKVKASASKQALVFQCVGCGAFHLQR

Trm1p   341  LGRISQREGRNNKTFTKYSVAQGPPVDTKCKFCEGTYHLAGPMYAGPLHNKEFIEEVLRI
Trmt1p  359  LGKASGVPSGR....AKFSAACGPPVTPECEHCGQRHQLGGPMWAEPIHDLDFVGRVLEA

Trm1p   401  NKEEHRDQDDTYGTRKRIEGMLSLAKNELSDSPFYFSPNHIASVIKLQVPPLKKVVAGLG
Trmt1p  415  VSANP....GRFHTSERIRGVLSVITEELPDVPLYYTLDQLSSTIHCNTPSLLQLRSALL

Trm1p   461  SLGFECSLTHAQPSSLKTNAPWDAIWYVMQKCDDEK.KDLSKMNPNTTGYKILSAMPGWL
Trmt1p  471  HADFRVSLSHACKNAVKTDAPASALWDIMRCWEKECPVKRERLSETSPAFRILSVEPRLQ

Trm1p   520  S...............GTVKSEYDSKLSFAPNEQSG........NIEKLRKLKIVRYQEN
Trmt1p  531  ANFTIREDANPSSRQRGLKRFQANPEANWGPRPRARPGGKAADEAMEERRRLLQNKRKEP

Trm1p   557  PTKNWGPKARPNTS..............................................
Trmt1p  591  PEDVAQRAARLKTFPCKRFKEGTCQRGDQCCYSHSPPTPRVSADAAPDCPETSNQTPPGP
```

Figure S1. Sequence comparison between Trm1p from *Saccharomyces cerevisiae* and Trmt1p from *Homo sapines*. Uniport entries of Trm1p and Trmt1p are P15565 and Q9NXH9, respectively. The overall identity and similarity between these two sequences are 34.2% and 50.4%, respectively. The alignments illustrated in Fig. S1-S8 were generated by the ClustalW web server using BLOSUM as the weight matrix [1] and these figures were prepared using ESPript [2].

```
Trm2p    1  MYEQFEFSFFFFENSDNKVKYKAHLISSIKRWSIITCMRCFWTVQKSIFKARFFACRNFV
RHO4a    1  ...........................................MNTLLFKRKGGNCG...

Trm2p   61  KKHNYKLISTMTGSTEMVPPTMKHTVDNKRLSSPLTDSGNRRTKKPKLRKYKAKKVETTS
RHO4a   15  ...NESNIVSQGSPSSSNLPESPGTLDEKNLPRLPTPFARSLSTIP...SYEQMKRTNKL

Trm2p  121  PMGVLEFEVNDLLKSQNLSREQVLNDVTSILNDKSSTDGPIVLQYHREVKNVKVLEITSN
RHO4a   69  PD..........................................YHLKIVVVGDGAVGKT

Trm2p  181  GNGLALIDNPVETEKKQVVIIPFGLPGDVVNIKVFKTHPYYVESDLLDVVEKSPMRRDDL
RHO4a   87  CLLISYVQGTFPTD.....YIPTIFENYVTNIEGPN........................

Trm2p  241  IRDKYFGKSSGSQLEFLTYDDQLELKRKTIMNAYKFFAPRLVAEKLLPPFDTTVASPLQF
RHO4a  118  ..........GQIIELALWDTAGQEEYSRLR.................PLSYTNADVLMV

Trm2p  301  GYRTKITPHFDMPKRKQKELSVRPPLGFGQKGRPQWRKDTLDIGGHGSILDIDECVLATE
RHO4a  151  CYSVGSKTSLKN..........VEDLWFP...............................

Trm2p  361  VLNKGLTNERRKFEQEFKNYKKGATILLRENTTILDPSKPTLEQLTEEASRDENGDISYV
RHO4a  170  ........EVKHFCPSTPIMLVGLKSDLYEADNLSDLVEP.................SSA

Trm2p  421  EVEDKKNNVRLAKTCVTNPRQIVTEYVDGYTFNFSAGEFFQNNNSILPIVTKYVRDNLQA
RHO4a  205  ESLAKRLGAFAHIQCSARLKENIDEVFETAIHTLLSDSLYAPREPTHTIKNPFKRN....

Trm2p  481  PAKGDDNKTKFLVDAYCGSGLFSICSSKGVDKVIGVEISADSVSFAEKNAKANGVENCRF
RHO4a  261  .......TTRSDIDSSTGDTSVSISGTKRLRKNK..........................

Trm2p  541  IVGKAEKLFESIDTPSENTSVILDPPRKGCDELFLKQLAAYNPAKIIYISCNVHSQARDV
RHO4a       ............................................................

Trm2p  601  EYFLKETENGSAHQIESIRGFDFFPQTHHVESVCIMKRI
RHO4a  288  .................................CIIM..
```

Figure S2. Sequence comparison between Trm2p from *Saccharomyces cerevisiae* and RHO4a protein from *Saccharomyces cerevisiae*. Uniport entries of Trm2p and RHO4a are P33753 and Q00246, respectively. The overall identity and similarity between these two sequences are 19.6% and 34.5%, respectively.

```
Trm3p       1  ................MVGGALICKYLPREEQLKLISDLIQNDSLEEVLELIETSPLDIT
TARBP1      1  MEWVLAEALLSQSRDPRALLGALCQGEASAERVETLRFLLQRLEDEEARGSGGAGALPEA

Trm3p      45  TDSNIETPIFEKITEQVIAYASIDG......EAREMFRSSRAEMNKALRTSAQLLCCLPS
TARBP1     61  AREVAAGYLVPLLRSLRGRPAGGPDPSLQPRHRRRVLRAAGAALRSCVRLAGRPQLAAAL

Trm3p      99  VWHKFQVWMSYRLNDIISENYKHLFNDN..FGKKIVQPFFDSFAEEQNANIK......HE
TARBP1    121  AEEALRDLLAGWRAPGAEAAVEVLAAVGPCLRPREDGPLLERVAGTAVALALGGGDGDE

Trm3p     151  NLHLDILSLLHYLEVVYLFDECKNGISSKCLDFIIVPLLGCNSEEIADSCSKLMRWHIKY
TARBP1    181  AGPAEDAAALVAGRLLPVLVQCGGAALRAVWGGLAAPGASLGSGRVEEKLLVLSALAEKL

Trm3p     211  LS..........KCCNTDSNFDKLIWTFIKQLYAEGSQQAWKQKNSLSFLLRFLLAAELS
TARBP1    241  LPEPGGDRARGAREAGPDARRCWRFWRTVQAGLGQADALTRKRARYLLQRAVEVSAELGA

Trm3p     261  PELITYIKTDAYWRHIQTELDNDVHEHRKLALSILKLTIQKLSSHGIT.....LQTTFYK
TARBP1    301  DCTCGPQEGNGPSLFWWSERKKDELLKFWENYILIMETLEGNQIHVIKPVLPKLNNLFEY

Trm3p     316  CNDLANIEMLGSWKKFTTLYEMIALDTSLNQIQAAKQDIIKIFDNEHLHHSWGLILLSTG
TARBP1    361  AVSEENGCWLFHPSWHMCIYKRMFESENKILSKEGVIHFLELYETKILPFSPEFSEFIIG

Trm3p     376  ......LKSSMESVRKYMMTLMFSITNMSAFSSNLPLLTKTLLPAAMSAHYFDVKGVSCP
TARBP1    421  PLMDALSESSLYSRSPGQPIGSCSPLGLKLQKFLVTYISLLPEEIKSSFLLKFIRKMTSR

Trm3p     430  HGEKLSLFVNNLLSQTTEGISDILFEILKLLVEKGTSFDPSRIYLSYGILVFFQNNKQKT
TARBP1    481  HWCAVPILFLSKALANVPRHKALGIDGLLALRDVIHCTMITHQILLRGAAQCYLLQTAMN

Trm3p     490  INSDHLSLIRKLYDFAAEEEVLETT..IQTIYLKFLLYIDPSVSASELLFTLVSHIKLKG
TARBP1    541  LLDVEKVSLSDVSTFLMSLRQEESLGRGTSLWTELCDWLRVNESYFKPSPTCSSIGLHKT

Trm3p     548  GTYKYVEPLFEDYR...............DLAVSHFDDLQAKENLTTNIGKDTIFDLLASI
TARBP1    601  SLNAYVKSIVQEYVKSSAWETGENCFMPDWFEAKLVSLMVLLAVDVEGMKTQYSGKQRTE

Trm3p     594  IFDFKDIDITPDFLIEVAKSKQDIPVYTSKAVTFLTQLLSGEPSNGYTYENATALLSYPN
TARBP1    661  NVLRIFLDPLLDVLMKFSTNAYMPLLKTDRCLQLLLKLLNTCRLKGSSAQDDEVSTVLQN
```

```
Trm3p    654  FTISTWKSINVNNLFKSVMEKFS....LDKFKFFAETYQKTYECRFDTIELNFNDLLSLY
TARBP1   721  FFMSTTESISEFILRRLTMNELNSVSDLDRCHLYLMVLTELINLHLKVGWKRGNPIWRVI

Trm3p    710  EMVKKSANQCSRESFKVKDSAYSSYFELLN.............................T
TARBP1   781  SLLKNASIQHLQEMDSGQEPTVGSQIQRVVSMAALAMVCEAIDQKPELQLDSLHAGPLES

Trm3p    741  FLKTYALNRDSSEGNDDELHILLRLVDENINKDNGN...........YLGNLAVCKLLYF
TARBP1   841  FLSSLQLNQTLQKPHAEEQSSYAHPLECSSVLEESSSSQGWGKIVAQYIHDQWVCLSFLL

Trm3p    790  IIDSYTHCST..........................SVSDDDIFIVKFIFEKFSFIWECI
TARBP1   901  KKYHTLIPTTGSEILEPFLPAVQMPIRTLQSALEALTVLSSDQVLPVFHCLKVLVPKLLT

Trm3p    824  NSERLVLKERDLHLMLIKGLFHPVILYFG.......SNQYIDTLTSKLEEHAQTIISLSY
TARBP1   961  SSESLCIESFDMAWKIISSLSNTQLIFWANLKAFVQFVFDNKVLTIAAKIKGQAYFKIKE

Trm3p    877  SRRSLLPLLGSQLRVFMKFYGKLLR...............EDVNYWWLINITVGVFKQPQ
TARBP1  1021  IMYKIIEMSAIKTGVFNTLISYCCQSWIVSASNVSQGSLSSAKNYSELILEACIFGTVFR

Trm3p    922  MDVNLYKLKPVISSLFDHKLNNYYIKGDELYEKVYGPDEILARVSIIDSILYANDQLKIR
TARBP1  1081  RDQRLVQDVQTFIENLGHDCAANIVMENTKREDHYVRICAVKFLCLLDGSNMSHKLFIED

Trm3p    982  LIEKVTEKTNALYAIKRTDGAEALQR..LLQWQLLLLSLLTTNEKKLSETSMIRILKSIE
TARBP1  1141  LAIKLLDKDELVSKSKKRYYVNSLQHRVKNRVWQTLLVLFPRLDQNFLNGIIDRIFQAGF

Trm3p   1040  DESSPLVRVYKEWFISSKVVDYYKTG.........NPKFAEDYLFSLLEDHSKPVFVVSA
TARBP1  1201  TNNQASIKYFIEWIIILILHKFPQFLPKFWDCFSYGEENLKTSICTFLAVLSHLDIITQN

Trm3p   1091  EKICFMVLKDLRNDEKKYGFTQLLDR...FICTLVPNAASNKPLVRHFSNSLIISLWPTF
TARBP1  1261  IPEKKLILKQALIVVLQWCFNHNFSVRLYALVALKKLWTVCKVLSVEEFDALTPVIESSL

Trm3p   1148  KAYLSDHTLRNIIENLYSNAKKTQIFGQYRAGDANTWDLKGDRKLTNMFGGVLKKVTDHD
TARBP1  1321  HQVESMHGAGNAKKNWQRIQEHFFFATFHPLKDYCLETIFYILPRLSGLIEDEWITIDKF

Trm3p   1208  CPYISESVFEKYLQEKDIVPIGTDERSLWLDKRD.TNTESVNNANISCDTSPLQTKSGAW
TARBP1  1381  TRFTDVPLAAGFQWYLSQTQLSKLKPGDWSQQDIGTNLVEADNQAEWTDVQKKIIPWNSR
```

```
Trm3p   1267 ETVLDLDNKKSNDVVTR....SELIVVSSLVDKPPNLGGICRLCDVLGVGLLTVQDIKVK
TARBP1  1441 VSDLDLELLFQDRAARLGKSISRLIVVASLIDKPTNLGGLCRTCEVFGASVLVVGSLQCI

Trm3p   1323 NHPQFKNVAVTADRWMPMEEVALDEIASFMKEKKKEGYTLIGLEQTDKSVKLDNNFQFPK
TARBP1  1501 SDKQFQHLSVSAEQWLPLVEVKPPQLIDYLQQKKTEGYTIIGVEQTAKSLDLT.QYCFPE

Trm3p   1383 KSLILLGTEAFGIPGTLLSELDLCLEIQQFGVIRSMNIQTATAVIVHSYTVQHM......
TARBP1  1560 KSLLLLGNEREGIPANLIQQLDVCVEIPQQGIIRSLNVHVSGALLIWEYTRQQLLSHGDT
```

```
Trm3p    121  HLFNDNFGKKIVQPFFDSFAEEQNANIKHENLHLDILSLLHYLEVVYLFDECKNGISSKC
TARBP2     1  .................MSEEEQGS.............................GTTTGC

Trm3p    181  LDFIIVPLLGCNSEEIADSCSKLMRWHIKYLSKCCNTDSNFDKLIWTFIKQLYAEGSQQA
TARBP2    15  GLPSIEQMLAANP...............................................

Trm3p    241  WKQKNSLSFLLRFLLAAELSPELITYIKTDAYWRHIQTELDNDVHEHRKLALSILKLTIQ
TARBP2        ............................................................

Trm3p    301  KLSSHGITLQTTFYKCNDLANIEMLGSWKKFTTLYEMIALDTSLNQIQAAKQDIIKIFDN
TARBP2    28  ..............GKTPISLLQEYGTRIGKTPVYDLLKAEG..................

Trm3p    361  EHLHHSWGLILLSTGLKSSMESVRKYMMTLMFSITNMSAFSSNLPLLTKTLLPAAMSAHY
TARBP2    56  .......................QAHQPNFTFRVT.........................

Trm3p    421  FDVKGVSCPHGEKLSLFVNNLLSQTTEGISDILFEILKLLVEKGTSFDPSRIYLSYGILV
TARBP2    68  ..VGDTSCTG..................................................

Trm3p    481  FFQNNKQKTINSDHLSLIRKLYDFAAEEEVLETTIQTIYLKFLLYIDPSVSASELLFTLV
TARBP2    76  .............................................QGPSKKAAKHKAAEV

Trm3p    541  SHIKLKGGTYKYVEPLFEDYRDLAVSHFDDLQAKENLTTNIGKDTIFDLLASIIFDFKDI
TARBP2    91  ALKHLKGG..SMLEPALEDS........................................

Trm3p    601  DITPDFLIEVAKSKQDIPVYTSKAVTFLTQLLSGEPSNGYTYENATALLSYPNFTISTWK
TARBP2   109  .........................SSFSPLDSSLPEDIPVFTAAAAATPVP........

Trm3p    661  SINVNNLFKSVMEKFSLDKFKFFAEIYQKTYECRFDTIELNFNDLLSLYEMVKKSANQCS
TARBP2   136  ....................................SVVLTRSPPMELQPPVSPQQSECN
```

```
Trm3p    721  RESFKVKDSAYSSYFELLNTFLKTYALNRDSSEGNDDELHILLRLVDENINKDNGNYLGN
TARBP2   160  .........PVGALQELVVQKG......................................

Trm3p    781  LAVCKLLYFIIDSYIHCSTSVSDDDIFIVKFIFEKFSFIWECINSERLVLKERDLHLMLI
TARBP2        ............................................................

Trm3p    841  KGLFHPVILYFGSNQYIDTLTSKLEEHAQTIISLSYSRRSLLPLLGSQLRVFMKFYGKLL
TARBP2        ............................................................

Trm3p    901  REDVNYWWLINIIVGVFKQPQMDVNLYKLKPVISSLFDHKLNNYYIKGDELYEKVYGPDE
TARBP2   173  ......WRLPEYTVTQESGP........................................

Trm3p    961  ILARVSIIDSILYANDQLKIRLIEKVTEKTNALYAIKRTDGAEALQRLLQWQLLLLSLLT
TARBP2        ............................................................

Trm3p   1021  TNEKKLSETSMIRILKSIEDESSPLVRVYKEWFISSKVVDYYKTGNPKFAEDYLFSLLED
TARBP2        ............................................................

Trm3p   1081  HSKPVFVVSAEKICFMVLKDLRNDEKKYGFTQLLDRFICTLVPNAASNKPLVRHFSNSLI
TARBP2   187  ......................AHRKEFTMTCRVERFIEIGSG...TSKKLAKRNAAAKM

Trm3p   1141  ISLWPTFKAYLSDHTLRNIIENLYSNAKKTQIFGQYRAGDANIWDLKGDRKLTNMFGGVL
TARBP2   222  LLRVHTVPLDARDGNEVEPDDDHFSIGVGSRLDGLRNRGPGCTWDS..............

Trm3p   1201  KKVTDHDCPYISESVFEKYLQEKDIVPIGTDERSLWLDKRDTNTESVNNANISCDTSPLQ
TARBP2   268  ..........LRNSVGEKILSLR.............................SCSLGSLG

Trm3p   1261  TKSGAWETVLDLDNKKSNDVVTRSELIVVSSLVDKPPNLGGICRLCDVLGVGLLTVQDIK
TARBP2   289  ALGPACCRVLSELSEE........QAFHVSYLDIEELSLSGLCQCLVELST.........

Trm3p   1321  VKNHPQFKNVAVTADRWMPMEEVALDEIASFMKEKKKEGYTLIGLEQTDKSVKLDNNFQF
TARBP2   332  ....................................QPATVCHGSATTREAARGEAARRA

Trm3p   1381  PKKSLILLGTEAFGIPGTLLSELDLCLEIQQFGVIRSMNIQTATAVIVHSYTVQHM
TARBP2   356  LQYLKIMAGSK.............................................
```

Figure S3. Sequence comparison between Trm3p from *Saccharomyces cerevisiae* and TRBP isoforms from *Homo sapiens*. Uniport entries of Trm3p, TARBP1, and TARBP2 are Q07527, Q13395, and Q15633, respectively. The overall identity and similarity between Trm3p and TARBP1 sequences are 15.9% and 33.4%, respectively. The overall identity and similarity between Trm3p and TARBP2 sequences are 21.9% and 39.9%, respectively.

```
Trm4p  121  PKTVIRKNEQFAKTQRFLVVENAVGNISRQEAVSMIPPIVLEVKPHHTVLDMCAAPGSKT
Pmt1     1  ..........MLSTKRLRVLELYSG.................IGGMHYALNLANIP....

Trm4p  181  AQLIEALHKDTDEPSGFVVANDADARRSHMLVHQLKRLNSANLMVVNHDAQFFPRIRLHG
Pmt1    30  ADIVCAIDINPQANEIYNLNHGKLAKHMDISTLTAKDFDAFDCKLWTMSPSCQPFTRIG.

Trm4p  241  NSNNKNDVLKFDRILCDVPCSGDGTMRKNVNVWKDWNTQAGLGLHAVQLNILNRGLHLLK
Pmt1    89  ...NRKDILDPR.............SQAFLNILNVLPHVNNLPEYILIENVQGFEESKAA

Trm4p  301  NNGRLVYSTCSLNPIENEAVVAEALRKWGDKIRLVNCDDKLPGLIRSKGVSKWPVYDRNL
Pmt1   133  EECRKVLRNCGYNLIEGILSPNQFN........IPNSRSRWYGLARLNFKGEWSIDDVFQ

Trm4p  361  TEKTKGDEGTLDSFFSPSEEEASKFNLQNCMRVYPHQQNTGGFFITVFEKVEDSTEAATE
Pmt1   185  FSEVAQKEGEVKRIR.............................................

Trm4p  421  KLSSETPALESEGPQTKKIKVEEVQKKERLPRDANEEPFVFVDPQHEALKVCWDFYGIDN
Pmt1   200  ......DYLEIERDWSSYMVLESVLNKWGHQFDIVKP.......................

Trm4p  481  IFDRNTCLVRNATGEPTRVVYTVCPALKDVIQANDDRLKIIYSGVKLFVSQRSDIECSWR
Pmt1   231  ..DSSSCCFTRG..............YTHLVQGAG............SILQMSDHENTHE

Trm4p  541  IQSESLPIMKHHMKSNRIVEANLEMLKHLLIESFPNFDDIRSKNIDNDFVEKMTKLSSGC
Pmt1   264  QFERNRMALQLRYFTAREVAR.........LMGFPESLEWSKSNVT..............

Trm4p  601  AFIDVSRNDPAKENLFLPVWKGNKCINLMVCKEDTHELLYRIFGIDANAKATPSAEEKEK
Pmt1   301  ...................................EKCMYRLLGNSINVKVVSYLISLLL

Trm4p  661  EKETTESPAETTTGTSTEAPSAAN
Pmt1   326  EPLNF...................
```

Figure S4. Sequence comparison between Trm4p from *Saccharomyces cerevisiae* and Pmt1

protein from *Schizosaccharomyces pombe*. Uniport entries of Trm4p and Pmt1 are P38205 and P40999, respectively. The overall identity and similarity between these two sequences are 17.9% and 37.0%, respectively.

```
Trm5p    1  MKIALPVFQKFNRLISSCKMSGVFPYNPPVNRQMRELDRSFFITKIPMCAVKFPEPKNIS
TrmD     1  ..MKIDILTLFPEMFSPLEHSIVG...........................KAREKGLLD

Trm5p   61  VFSKNFKNCILRVPRIPHVVKLNSSKPKDELTSVQNKKLKTADGNNTPVTKGVLLHESIH
TrmD    32  IQYHNFR...ENAEKARHVDDE.........................PYGGGQGMLLRAQ

Trm5p  121  SVEDAYGKLPEDALAFLKENSAEIVPHEYVLDYDFWKAEEILRAVLPEQFLEEVPTGFTI
TrmD    64  PIFDSFDAIEKKNPRVILLD................PAGKQFDQAYAEDLAQEEELIFIC

Trm5p  181  TGHIAHLNLRTEFKPFDSLIGQVILDKNNKIECVVDKVSSIATQFRTFPMKVIAGKSDSL
TrmD   108  G..........HYEGYDERIKTLVTDEISLGDYVLTGGELAAMTMIDATVRLIPEVIGKE

Trm5p  241  VVEQKESNCTFKFDFSKVYWNSRLHTEHERLVKQYFQPGQVVCDVFAGVGPFAVPAGKKD
TrmD   158  SSHQDDS.............FSSGLLEYPQYTRPYDYRGMVVPDVLMSG...........

Trm5p  301  VIVLANDLNPESYKYLKENIALNKVAKTVKSFNMDGADFIRQSPQLLQQWIQDEEGGKIT
TrmD   194  ..........................................HHEKIRQWRLYES.....

Trm5p  361  IPLPLKKRHRSQQHNDQQPPQPRTKELIIPSHISHYVMNLPDSAISFLGNFRGIFAAHTK
TrmD   207  LKKTYERR.....................PDLLEHYQLTVEE..................

Trm5p  421  GATDTIQMPWVHVHCFEKYPPGDQVTEDELHARVHARIIAALKVTADDLPLNAVSLHLVR
TrmD   228  ...................................EKMLAEIKENKE.............
```

Figure S5. Sequence comparison between Trm5p from *Saccharomyces cerevisiae* and TrmD from *Streptococcus pneumoniae*. Uniport entries of Trm5p and TrmD are P38793 and B8ZND2, respectively. The overall identity and similarity between these two sequences are 17.6% and 38.9%, respectively.

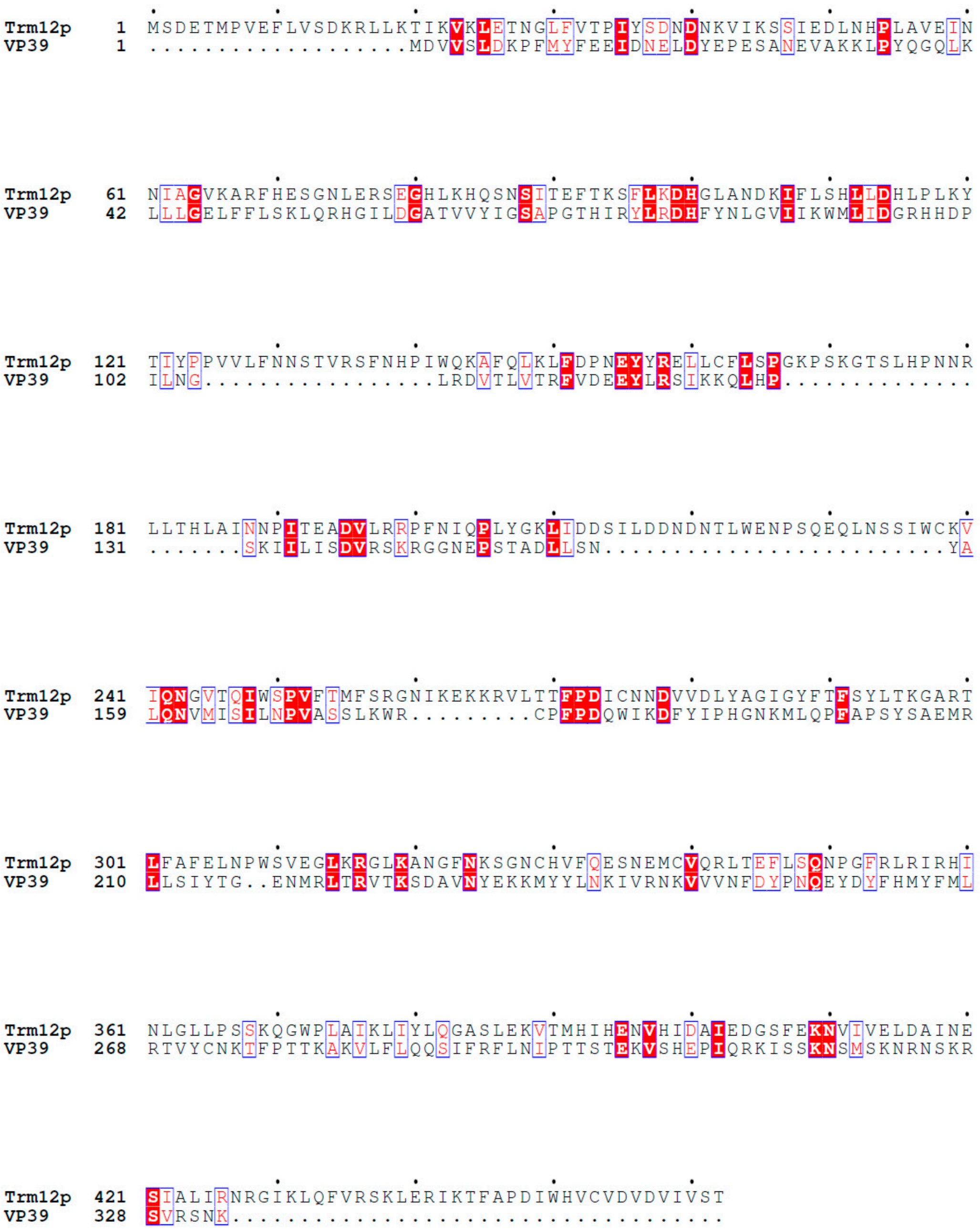

Figure S6. Sequence comparison between Trm12p from *Saccharomyces cerevisiae* and VP39 protein from *Vaccinia virus*. Uniport entries of Trm12p and VP39 are Q04235 and P07617, respectively. The overall identity and similarity between these two sequences are 14.4% and 27.6%, respectively.

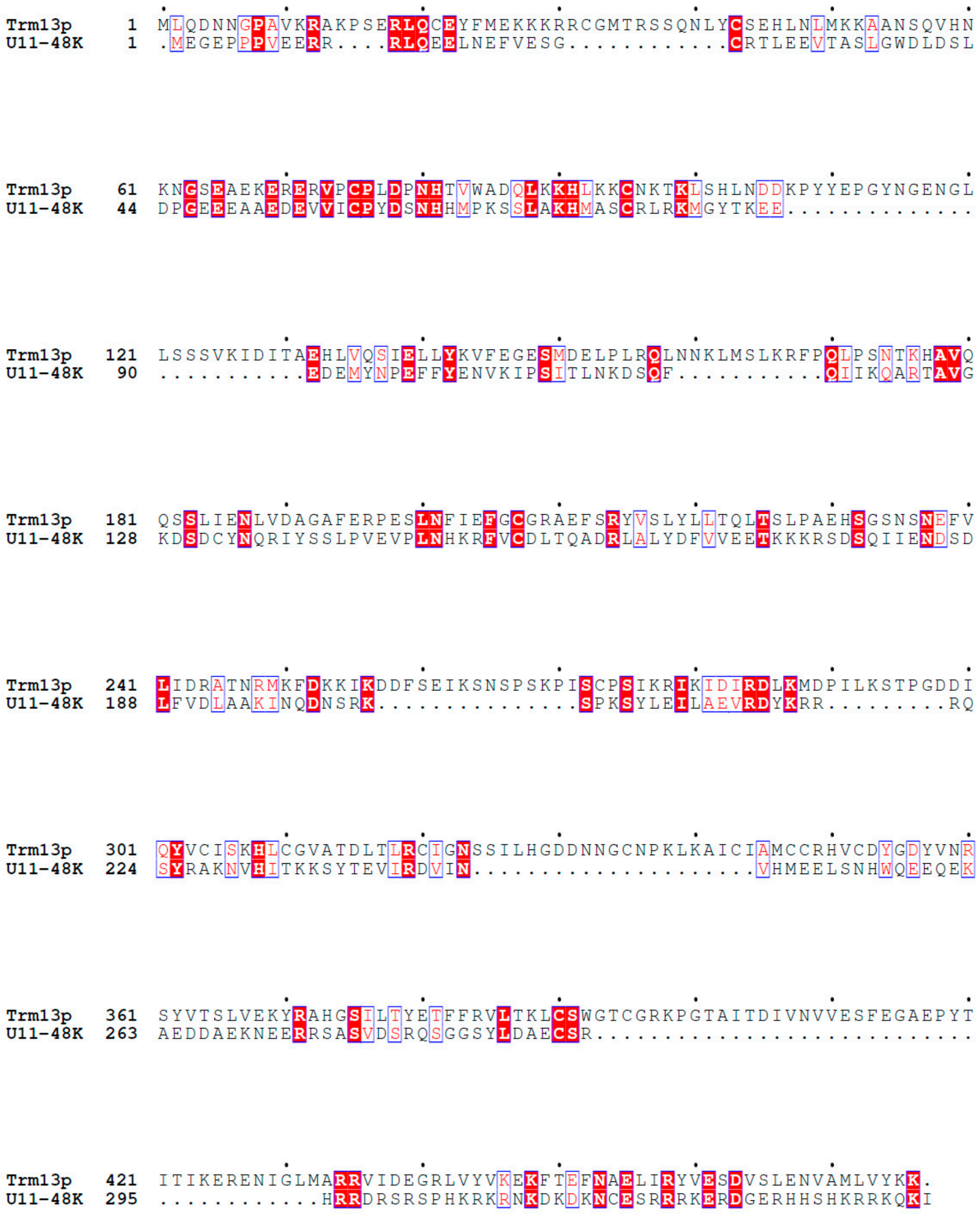

Figure S7. Sequence comparison between Trm13p from *Saccharomyces cerevisiae* and U11-48K protein from *Homo sapiens*. Uniport entries of Trm13p and U11-48K are Q12383 and Q6IEG0, respectively. The overall identity and similarity between these two sequences are 19.8% and 31.6%, respectively.

```
Trm44p     1  MTGDGSAHISKNNQNQHKDRFKFIVNDKS....ILGPQWLSLYQTDGKVTFAKSHFEQAM
Trmt44p    1  MAEVGRTGISYPGALLPQGFWAAVEVWLERPQVANKRLCGARLEARWSAALPCAEARGPG

Trm44p    57  MNVIREPNINSTVILRADILKEINHAAEAGSEPKFDESVLKKFEIDNGNESGEEDVKKIN
Trmt44p   61  TSAGSEQKERGPGPGQGSPGGGPGPRSLSGPEQGTACCELEEAQGQCQQEEAQREAASVP

Trm44p   117  IEDLNIR.....SCETSESLKLSPVHEFVRRIIPRNFYKDAIINQTCLILNSKDPNFQET
Trmt44p  121  LRDSGHPGHAEGREGDFPAADLDSLWEDFSQSLAR.GNSELLAFLTSSGAGSQPEAQREL

Trm44p   172  SLIVYTPHINSEKDCPFYIPRTQSVGILLHQSVLSVHYIPFPEDKTAFTDES........
Trmt44p  180  DVVLRTVIPKTSPHCPLTTPRREIVVQDVLNGTITFLPLEEDDEGNLKVKMSNVYQIQLS

Trm44p   224  .......................ERVVRTAYRLLQTANKHSKGVMQGYEKRVNHDQVVNKV
Trmt44p  240  HSKEEWFISVLIFCPERWHSDGIVYPKPTWLGEELLAKLAKWSVENKKSDFKSTLSLISI

                                     Motif X
Trm44p   262  NFQNTYIVLKKKYSKFLVENWAESTDPKKHVFEDIAIAAFLIELWIKVYGPDFRSKMQ.F
Trmt44p  300  MKYSKAYQELKEKYKEMVKVWPEVTDPEKFVYEDVAIAAYLLILWEEERAERRLTARQSF

                Motif I          Motif II         Motif III
Trm44p   321  RDLGCGNGALCYILLSESIKGLGIDARKRKSWSIYPPEVQSSLKEQVIIPSILLRPHPAL
Trmt44p  360  VDLGCGNGLLVHILSSEGHPGRGIDVRRRKIWDMYGPQTQ..LEEDAITPNDKTLFPDVD

Trm44p   381  KRQVPHLEHNGRFFPVKVTHEVIAPATVVYSSEDLLKSPQVN............TAEFPP
Trmt44p  418  WLIGNHSDELTPWIPVIAARSSYNCRFFVLPCCFFDFIGRYSRRQSKKTQYREYLDFIKE

                Motif IV
Trm44p   429  DTFIIGNHSDELTCWIP......LLGHPYMVIPCCSHNFSGQRVRFNVRKR.........
Trmt44p  478  VGFTCGFHVDEDCLRIPSTKRVCLVGKSRTYPSSREASVDEKRTQYIKSRRGCPVSPPGW

Trm44p   474  ..................................................SPRSNEIK
Trmt44p  538  ELSPSPRWVAAGSAGHCDGQQALDARVGCVTRAWAAEHGAGPQAEGPWLPGFHPREKAER

Trm44p   482  NQNNSKSTYSGLVDHVEYISSRVGWKVEKEMLRIPSTRNAAIIGVENATLKHFP....TQ
Trmt44p  598  VRNCAALPRDFIDQVVLQVANLLLGGKQLNTRSSRNGSLKTWNGGESLSLAEVANELDTE

Trm44p   538  AVYDMIWEDGGAEGWIQNTMSLLKRN..................................
Trmt44p  658  TLRRLKRECGGLQTLLRNSHQVFQVVNGRVHIRDWREETLWKTKQPEAKQRLLSEACKTR

Trm44p   564  .....................PRNH.................
Trmt44p  718  LCWFFMHHPDGCALSTDCCPFAHGPAELRPPRTTPRKKIS
```

Figure S8. Sequence comparison between Trm44p from *Saccharomyces cerevisiae* and Trmt44p from *Homo sapiens*. Uniport entries of Trm44p and Trmt44p are Q02648 and Q8IYL2, respectively. The overall identity and similarity between these two sequences are 18.1% and 32.3%, respectively. The motifs conserved in DUF1613 proteins are indicated above the sequences.

References

[1] Thompson, J.D., Gibson, T.J., Higgins, D.G., 2002. Multiple sequence alignment usingClustalW and ClustalX. Curr. Protoc. Bioinf. Chap. 2, Unit 2.3.

[2] Robert, X., Gouet, P., 2014. Deciphering key features in protein structures with the new ENDscript server. Nucleic Acids Res. 42, W320-324.
